# Cyberchondria and health anxiety among Tunisian university students: A cross-sectional study

**DOI:** 10.1192/j.eurpsy.2026.12066

**Published:** 2026-07-31

**Authors:** I. Chaari, H. Guidara, Z. Hadrich, M. Kessentini, W. Abid, E. Maaloul, A. Kammoun, F. Charfeddine, L. Aribi, N. Messedi, J. Aloulou

**Affiliations:** 1 Psychiatric department “B”, Hedi Chaker University Hospital; 2 Higher institute of nursing sciences of Sfax, Sfax, Tunisia

## Abstract

**Introduction:**

Cyberchondria, defined as excessive and repetitive online searching for health-related information, is an emerging challenge in mental health. It may aggravate health anxiety, reduce quality of life, and increase healthcare utilization. While studied in other regions, little evidence exists from North Africa.

**Objectives:**

To assess the prevalence of cyberchondria, health anxiety, Internet addiction, and eHealth literacy among Tunisian university students.

**Methods:**

We conducted a cross-sectional online survey among university students in Sfax (Tunisia) between March and April 2025. Validated instruments were used: Cyberchondria Severity Scale-15 (CSS-15), Short form of Health Anxiety Inventory (SHAI), Internet addiction test (IAT), and eHealth literacy scale (eHEALS). Sociodemographic and clinical data included gender, academic year, and psychiatric history.

**Results:**

A total of 283 Tunisian students participated (58.7% were male, mean age 21.32 ± 1.41 years). Cyberchondria was moderate in 64% and high in 3.9%. Health anxiety was moderate/high in 8.8% and substantial in 7.4%. Internet addiction was moderate in 53.7% and severe in 13.4%. eHealth literacy was low in 44.5% and high in 16.6%. Cyberchondria was higher in females (p<0.001), in those with family psychiatric history (p<0.001), and in students with Internet addiction (ρ=0.22, p<0.001).

**Conclusions:**

Cyberchondria is highly prevalent among Tunisian university students, strongly linked to health anxiety and problematic Internet use. Preventive interventions should include psychoeducation and digital health literacy programs.

**Disclosure of Interest:**

None Declared

